# Fish – a scoping review for Nordic Nutrition Recommendations 2023

**DOI:** 10.29219/fnr.v68.10485

**Published:** 2024-03-05

**Authors:** Johanna E. Torfadottir, Stine M. Ulven

**Affiliations:** 1 Centre of Public Health Sciences, University of Iceland, Reykjavik, Iceland; 2 Directorate of Health, Reykjavik, Iceland; 3 Department of Nutrition, University of Oslo, Oslo, Norway

**Keywords:** fish, seafood, polyunsaturated fatty acids, dietary recommendations

## Abstract

The aim of this scoping review was to conduct evidence-based documentation between fish intake and health outcomes for food-based dietary guidelines (FBDGs) in the Nordic Nutrition Recommendations (NNR) 2023. For most health outcomes, the evidence for fish oil and n-3 long chain (LC) polyunsaturated fatty acids (PUFA) supplementation was included when examining evidence between fish intake and health. In this review, conclusions from qualified systematic reviews (qSR) approved by NNR2023 are included. In addition, conclusions of a *de novo* systematic reviews on the topic of n-3 LC-PUFA, asthma, and allergy are included. Finally, a systematic literature search was performed limited to systematic reviews and meta-analysis published between 2011 and September 2021. In total, 21 papers from the systematic literature search, four qSR, and eight reports were included addressing the association between fish intake, fish oil, and n-3 LC-PUFA supplementation on several health outcomes. These included cardiovascular disease (CVD), type 2 diabetes, cancers (colorectal, breast, and prostate), metabolic syndrome, obesity, mortality, cognition and mental health, pregnancy-related outcomes (preterm birth and birth weight), and outcomes specific for children (neurodevelopment, and risk of food allergies, and asthma). In addition, intermediate risk factors such as blood lipids, glucose, C-reactive protein, and blood pressure were reviewed. Based on current evidence, fish consumption can have beneficial effects to prevent coronary heart disease (CHD) and stroke incidence, and lower mortality from CVD, CHD, myocardial infarction (MI), and stroke, as well as total mortality risk. In addition, fish consumption is beneficial for preventing cognitive decline in adults (e.g. dementia and Alzheimer’s disease). Fish intake may also prevent metabolic syndrome, supported by an observed association between fish intake and reduction in plasma triglycerides and increase in high-density lipoprotein (HDL) cholesterol levels. Data from fish oil and n-3 LC-PUFA supplementation studies supports the conclusions on the effects of fish consumption on most of the health outcomes.

## Popular scientific summary

- Fish is an important part of the Nordic diet, and is a good source of several important nutrients, such as vitamin D, vitamin B_12_, and iodine.
- Health effects of fatty fish have particularly been attributed to the content of omega-3 polyunsaturated fatty acids.
- The evidence for protective associations between fish intake and total mortality, cardiovascular disease and cognitive decline is considered as strong.
- Maternal fish consumption during pregnancy probably has a beneficial effect on birth outcomes.
- Evidence regarding effects of fish intake on risk of type 2 diabetes or cancer is limited.
- Fatty fish may contain environmental contaminants, but the benefits of eating fish outweigh the potential risks.

Fish is a substantial part of the Nordic diet (1), and contains important nutrients for optimal function of the body. The fat content in fish varies greatly between species, and thus the nutrient content differs. Lean fish, such as cod, haddock, saithe, plaice, and pike, contain less than 2 g of fat per 100 g. However, a high proportion of the fatty acids in lean fish is n-3 long-chain (LC) polyunsaturated fatty acids (PUFA) (2). The content of fat in medium fatty fish, such as winter-mackerel, halibut, catfish, and tuna, is 2–8 g of fat per 100 g. Fatty fish, such as herring, summer-mackerel, trout, salmon, and eel, contain more than 8 g of fat per 100 g. Medium-fat and fatty fish are the major dietary sources of the n-3 LC-PUFA eicosapentaenoic acid (EPA) and docosahexaenoic acid (DHA). Fish also contains monounsaturated and saturated fatty acids (MUFA and SFA), including odd-chain fatty acids (e.g. C15:0 and C17:0) (3). Fatty fish is a major source of dietary vitamin D, and some species contain vitamin A (retinol) (3). In addition, cod liver contains high amounts of n-3 LC-PUFA, vitamin D, and vitamin A (retinol). Fish in general is also a good source of protein, vitamin B_12_, iodine, and selenium. In addition, the nutrient content might vary between types of fish, wild fish, and farmed fish. Dairy products, meat, and fatty fish can contain environmental toxins, such as methyl mercury, and persistent organic pollutants (POPs) such as polychlorinated-p-dioxins (PCDDs) and the related furans (PCDFs), and dioxins and dioxin-like polychlorinated biphenyls (DL-PCBs). In general, fish captured in the open sea has lower concentrations of pollutants than fish from the Baltic Sea or Norwegian fjords. Some marine fish (e.g. large tuna and halibut – higher in the food chain) and freshwater fish from certain areas might contain elevated levels of methyl mercury. As an example, the mean range (mg/kg wet weight) of total mercury in lean fish in Norway is 0.06–0.12, and in fatty fish is 0.02–0.19 (4). Lean fish generally contains lower levels of POPs, and in Norway, the mean range of PCDD/PCDF/DL-PCBs in different species of lean fish is 0.064–0.594 pg/g, and in different species of fatty fish the mean range of these POPs is 0.488–2.39 pg/g (4). Due to this knowledge, all the national food agencies of the Nordic countries have issued specific advice on fish consumption for specific population groups (i.e. children and women of fertile age) (3).

The aim of this scoping review is to describe the totality of evidence based on qualified systematic reviews (qSRs) defined by the Nordic Nutrition Recommendations 2023 (NNR2023), for the role of fish consumption for health-related outcomes as a basis for setting food-based dietary guidelines (FBDGs) (Box 1).

*Box 1.* Background papers for Nordic Nutrition Recommendations 2023

- This paper is one of many scoping reviews commissioned as part of the Nordic Nutrition Recommendations 2023 (NNR2023) project (6).
- The papers are included in the extended NNR2023 report but, for transparency, these scoping reviews are also published in Food & Nutrition Research.
- The scoping reviews have been peer reviewed by independent experts in the research field according to the standard procedures of the journal.
- The scoping reviews have also been subjected to public consultations (see report to be published by the NNR2023 project).
- The NNR2023 committee has served as the editorial board.
- While these papers are a main fundament, the NNR2023 committee has the sole responsibility for setting dietary reference values in the NNR2023 project.

## Methods

To describe the totality of evidence for the role of fish consumption for health-related outcomes, existing qSRs defined by the NNR2023 (5, 6), an initial literature search by the NNR2023 committee (7, 8), a *de novo* SR on the topic n-3 LC-PUFA, asthma and allergy (9, 10), and a benefit and risk assessment of seafood consumption by the Norwegian Scientific Committee for Food and Environment (VKM) (4) was included. In addition, four qSR reviews (7–9, 11) and eight reports (4, 12–18) (including the VKM report) were included. All the qSRs adhere to the eligibility criteria determined by the NNR2023 project (6). The outcomes included in these qSRs were cardiovascular disease (CVD), type 2 diabetes (T2D), obesity, cancer, mortality, and pregnancy-related outcomes (preterm birth and birth weight), neurodevelopment in children, cognition and mental health in adults, and food allergies and asthma in children (Table 1). In addition, a scoping review was conducted in accordance with the protocol developed within the NNR2023 project (6). The literature search was conducted in PubMed/MEDLINE using the search string (fish*[Title/Abstract] OR seafood*[Title/Abstract] OR fish oil*[Title/Abstract] AND (2011:2021[pdat]) AND humans[Filter] AND (meta-analysis[Filter] OR systematicreview[Filter]) AND english[Filter]. The search was limited to systematic reviews and meta-analysis of human data published since 2011 to September 2021. The outcomes of interests in the current review were intermediate CVD risk factors, including lipids (total cholesterol, low-density lipoprotein (LDL)-cholesterol, HDL-cholesterol, triglycerides), blood pressure, and the inflammatory marker C-reactive protein, obesity (fish oil only since fish consumption was covered in qSR), metabolic syndrome, and some cancers (colorectal, breast, and prostate) common in the Nordic countries.

**Table 1:** Fish consumption and conclusions of relevant health outcomes from qSR defined by NNR2022

Report	Health outcome	Conclusion	Comment
	**Obesity and body weight**		
VKM2022	Overweight in adults	Limited, no conclusion	Protocol approved by NNR2022
VKM2022	Overweight in children	Limited, no conclusion	Protocol approved by NNR2022
	**CVD outcomes**		
VKM2022	CHD incidence	Probably protective	Protocol approved by NNR2022
VKM 2022	Stroke incidence	Probably protective	Protocol approved by NNR2022
VKM 2022	CVD mortality	Probably protective	Protocol approved by NNR2022
VKM 2022	CHD mortality	Probably protective	Protocol approved by NNR2022
VKM 2022	MI mortality	Probably protective	Protocol approved by NNR2022
VKM 2022	Stroke mortality	Probably protective	Protocol approved by NNR2022
VKM 2022	CVD incidence	Limited, suggestive protective	Protocol approved by NNR2022
VKM 2022	MI incidence	Limited, suggestive protective	Protocol approved by NNR2022
VKM 2022	Heart failure	Limited, suggestive protective	Protocol approved by NNR2022
VKM 2022	Atrial fibrillation	Limited, suggestive adverse	Protocol approved by NNR2022
American Heart association (Rimm EB et al 2018)	Fatal and nonfatal CHD events, including sudden cardiac death	Strongly associated with lower risk	qSR identified by NNR based on original search by the NNR committee
American Heart association (Rimm EB et al 2018)	Heart failure	Heterogeneity in findings, protective or no association	qSR identified by NNR based on original search by the NNR committee
American Heart association (Rimm EB et al 2018)	CHD incidence	Modestly associated with lower risk	qSR identified by NNR based on original search by the NNR committee
American Heart association (Rimm EB et al 2018)	Stroke	Associated with lower risk of stroke (ischemic)	qSR identified by NNR based on original search by the NNR committee
DGAC (USA) 2020	CVD risk and intake during childhood and adolescence	No conclusions due to few studies and serious methodological limitations of the included studies.	qSR identified in Høyer et al 2021
VKM 2022	**Type 2 diabetes**	Limited, no conclusion	Protocol approved by NNR2022
	**Cancer**		
VKM 2022	Colorectal cancer	Limited, suggestive protective	Protocol approved by NNR2022
WCRF 2018	Colorectal cancer	Limited, suggestive protective	qSR identified in Høyer et al 2021
	**Neurodevelopment**		
VKM 2022	Child neurodevelopment (maternal exposure)	Limited, suggestive protective	Protocol approved by NNR2022
VKM 2022	Child neurodevelopment (exposure in children)	Limited, suggestive protective	Protocol approved by NNR2022
DGAC (USA) 2020	Neurocognitive development (maternal exposure)	Moderate evidence for favorable assocation with measures of cognitive development. Limited evidence for language and communication development. Insufficient evidence for movement and physical development, social-emotional and behavioral development in the child. No studies met inclusion criteria maternal seafood intake during lactation and neurocognitive development	qSR identified in Høyer et al 2021
DGAC (USA) 2020	Neurocognitive development (exposure during childhood and adolescence)	Insufficient evidence	qSR identified in Høyer et al 2021
	**Cognition and mental health**		
VKM 2022	Cognitive decline in adults (e.g. dementia and alzheimer’s disease	Probable protective	Protocol approved by NNR2022
VKM 2022	Mental health in adults (depression)	Limited, suggestive protective	Protocol approved by NNR2022
VKM 2022	Postpartum depression	Limited, suggestive protective	Protocol approved by NNR2022
	**Asthma and allergies**		
VKM 2022	Asthma-maternal exposure)	Limited, no conclusion	Protocol approved by NNR2022
VKM 2022	Wheeze (maternal exposure)	Limited suggestive first 2 years of life and Limited, no conclusion older age	Protocol approved by NNR2022
VKM 2022	Allergic sensitization	Limited, no conclusion	Protocol approved by NNR2022
	**Birth outcomes**		
VKM 2022	Preterm birth	Probably protective	Protocol approved by NNR2022
VKM 2022	Low birth weight	Probably protective	Protocol approved by NNR2022
VKM 2022	**All-cause mortality**	Probably protective	Protocol approved by NNR2022

These outcomes were based on existing qSR (5) and a benefit and risk report of seafood consumption by Norwegian Scientific Committee for Food and Environment (VKM) that is approved by NNR2022 as qSR based on published protocol. The VKM report is a systematic literature review based on a systematic literature search, quality assessment of the identified literature, and a weight of evidence approach that follows the guidelines described by the World Cancer Research Fund (18). Probable (strong evidence) is defined as evidence strong enough to support a judgement of probably causal relationship, which generally justifies recommendations designed to reduce the risk of an outcome. Limited, suggestive, is defined as evidence that is too limited to permit a probably or convincing causal judgement, but is suggestive of a direction of effect. Limited, no conclusion is defined as evidence is so limited that no firm conclusions can be made.

The PubMed search in September 2021 resulted in 856 publications. Based on the abstracts and the outcomes of interest, updates, and duplicates removal, 37 reviews were read in full text. Both authors independently used Rayyan (https://rayyan.ai) as a tool to screen all abstracts for eligibility. After the blinding was removed, we agreed on selection of reviews to include. Disagreement regarding inclusion or exclusion of a review was solved by discussions between the authors. Sixteen reviews were excluded due to different factors such as being conducted in Asia, where there are different fish species, possible pollution in lakes, and different preservation methods. Other reasons for exclusions were outcomes already covered by defined qSRs, outcomes not within the scope of this scoping review, use of biomarkers instead of dietary data, and having dietary pattern as an exposure, making it challenging to examine the effect of fish intake alone. In total, 21 reviews were included in the scoping review, and the results are presented in Tables 2 to 8. All sources of evidence considered in this scoping adhere to the eligibility criteria determined by the NNR2023 project (19–21).

**Table 2:** Characteristics of the studies evaluating n-3 LC-PUFA supplementation and fish oil supplementation and body weight

Author	Outcomes	Type of study	Exposure	Results	Conclusion
**Du et al., 2015** PlosOne	Some parameters of body composition in overweigt/obese adults	SR and MA 21 RCTs (*n* = 1,652)	Fish oil or marine food (one trial) Median dose of n-3 LC-PUFA was 1.92 g/day (range 0.54–11.3 g/day), median duration 12 weeks (range 4–24 weeks)	No effect on reducing body weight, SMD -0.07 (95% CI -0.21 to 0.07) and BMI, SMD -0.09 (95% CI -0.22 to 0.03). No significant waist circumference reduction, SMD -0.14 (95% CI -0.36 to 0.06) Waist hip ratio significantly reduced, SMD-0.39 (95% CI -0.72 to -0.06)	No anti-obesity effect of n3 LC-PUFA
**Jazayeri et al., 2020** Complementary therapies in medicine	Antropometric indices in children and adolescents	SR and MA 6 RCTs (*n* = 342)	n-3 LC-PUFA supplementation or fish oil supplementation n-3 LC-PUFA dose ranged 250–3360 mg/day, 8–24 weeks	No significant differences in body weight reduction, SMD -0.00 (95% CI -0.26 to 0.25) No significant reduction in BMI, SMD -0.07 (95% CI -0.32 to 0.17) No significant waist circumference reduction, SMD -0.16 (95% CI -0.51 to 0.19)	n-3 LC-PUFA supplementation does not change antropometric indices in children and adolescents.

SR, systematic review; MA, meta-analysis; SMD, standard deviation mean difference.

**Table 3:** Characteristics of the studies evaluating fish intake, n-3 LC-PUFA intake and fish oil supplements and hypertension

Author	Outcomes	Type of study	Exposure	Results	Conclusion
**Campbell et al., 2012** Eur J Preventive cardiology	Effectiveness of fish-oil supplementation on BP	Systematic review of RCTs 17 RCTs *N* = 1,524 Normotensive and hypertensive participants (at least BP 140/85 mmHg)	Fish oil supplementation, range EPA+DHA; 0.8–5.08 g/day	8 studies in hypertensive participants found a statistically significant reduction in SBP; 2.56 mmHg (95% CI 0.58 to 4.53) and in DBP; 1.47 mmHg (95% CI 0.41 to 2.53) 9 studies in normotensive participants showed a non-significant reduction in both SBP and DBP.	Small reduction in BP among hypertensive participants
**Miller et al., 2014** Am J Hypertension	Examine the effect of EPA+DHA, without upper dose limits and including food sources on blood pressure	MA of RCTs 70 RCTs included	Fish oil supplementation, range EPA-DHA; 0.1–15.0 g/day	Compared to placebo, EPA+DHA reduced SBP -1.52 mmHg (95% CI -2.25 to -0.79) and DBP -0.99 mmHg (95% CI -1.54 to -0.44) The strongest effect were observed among untreated hypertensive subjects, although blood pressure was lowered among normotensive subjects	Provision of EPA-DHA may reduce SBP and provision of ≥2 g reduced DBP.
**Yang et al., 2016** Nutrients	Elevated blood pressure Defined as SBP≥140 mm Hg or DBP≥90 mm Hg in four studies and as SBP≥130 mm Hg or DBP≥85 mm Hg in four studies	MA 8 cohort studies *N* = 56,204 adults and *n* = 20,497 elevated blood pressure events Follow-up from 3 to 20 years	Comparing highest versus lowest fish intake, 4 cohort studies, *n* = 17,710 and *n* = 3,590 cases Comparing highest versus lowest n-3 LC-PUFA intake , 6 cohort studies, (three studies for biomarkers and three studies for diet), *n* = 38,494 and n = 16,907 cases Comparing highest versus lowest n-3 LC-PUFA intake, 3 cohort studies, (studies for diet), n = 36,112 and *n* = 15,245 cases	Highest versus lowest intake and RR (95% CI) for hypertension 0.96 (0.81 to 1.14), *I*^2^ = 44.7%; *P*-heterogeneity = 0.143 Each 20 g/day increment of fish consumption was not significantly associated with reduced risk of elevated BP RR 0.98 (0.94 to 1.03) Highest versus lowest n-3 LC-PUFA category and RR (95% CI) for hypertension 0.73 (0.60 to 0.89), *I*^2^ = 75%; *P*-heterogeneity = 0.001 Highest versus lowest n-3 LC-PUFA intake and RR (95% CI) for hypertension 0.80 (0.58 to 1.10), *I*^2^ = 79.3%; *P*-heterogeneity = 0.002	No association between fish or dietary n-3 LC-PUFA intake and incidence of elevated BP. Circulating n-3 LC-PUFA as biomarkers of dietary intake are inversely associated with incidence of elevated BP. No association between high and low intake of n-3 LC-PUFA
**Schwingshackl et al., 2017** Adv Nutr	Risk of hypertension The incidence of hypertension was defined as SBP≥140 mm Hg or DBP≥90 mm Hg for the first time in any follow-up checkup or when taking antihypertensive medication for the first time in the follow-up visits	SR and MA 8 prospective cohort, case-cohort, and nested case–control studies, and follow-ups of RCTs *N* = 83,612 incident cases	Comparing extreme fish intake categories (range of intake 0–156 g/day)	Highest versus lowest intake and RR (95% CI) for hypertension 1.01 (0.92 to 1.10), *I* ^2^ = 57%; *P*-heterogeneity = 0.02 An increase in fish intake by 100 g/day and RR (95% CI) for hypertension 1.07 (0.98 to 1.16), *I*^2^ = 74%; *P*-heterogeneity < 0.0001 Nonlinear dose-response association (P-nonlinearity < 0.01; *n* = 7 studies) Risk increased by 8% with increasing intake of fish ≤100 g/day	No association between high and low intake of fish, and dose-response Fish consumption was associated with a slight increase in hypertension risk in the nonlinear dose-response analysis.
**Alhassan et al., 2017** Atherosclerosis	Vascular risk factors. Primary outcomes included lipid biomarkers (TG, TC, HDL-C, LDL-C, VLDL-C, SBP, DBP, glucose, insulin, HOMA-IR, inflammation markers	SR and MA 14 clinical trials and RCTs *N* = 1,378 adults (>18 years) Mean follow up for 9 weeks (4–24 weeks)	Fish intake-the frequencies for consuming fish ranged from once a week to daily consumption, and portion size of oily fish (most commonly salmon), consumed on a given day ranged from 20 to 500 g.		No effect of fish consumption on blood pressure
**Abdelhamid et al., 2020** Cochrane Database Syst Rev	TC TG HDL-C LDL-C DBP SBP	SR and MA 30 RCTs 28 RCTs 30 RCTs 25 RCTs 15 RCTs 17 RCTs	Fish oil supplementation (mainly), dietary intake Comparing high versus low long chain n-3 fatty acid intake	No effect on DBP mean difference -0.02 (95% CI -0.22 to 0.17) No effect on SBP mean difference 0.01 (95% CI -0.31 to 0.34)	No effect of fish oil supplementation on DBP and SBP

BP, blood pressure; SBP, systolic blood pressure; DBP, diastolic blood pressure; SR, systematic review; MA, meta-analysis; RCT, randomized controlled trial; HR, Hazard ratio; OR, Odds ratio; RR, Relative risk/Risk ratio.

**Table 4:** Characteristics of the studies evaluating fish intake and fish oil supplements and lipids

Author	Outcomes	Type of study	Exposure	Results	Conclusion
**Alhassan et al., 2017** Atherosclerosis	Vascular risk factors. Primary outcomes included lipid biomarkers (TG, TC, HDL-C, LDL-C, VLDL-C, SBP, DBP, glucose, insulin, HOMA-IR, inflammation markers	SR and MA 14 clinical trials and RCTs *N* = 1378 adults (>18 years) Mean follow up for 9 weeks (4–24 weeks)	Fish intake-the frequencies for consuming fish ranged from once a week to daily consumption, and portion size of oily fish (most commonly salmon), consumed on a given day ranged from 20 to 500 g.	Interventions on oily fish consumption decreases the levels of TG significantly (mean difference -0.11 mmol/L), 95% CI -0.18 to -0.04 (*P* = 0.002) compared to control group Interventions on oily fish consumption increase the levels of HDL-C significantly (mean difference 0.06 mmol/L), 95% CI 0.02 to 0.11 (*P* = 0.008) compared to control group No effect on TC and LDL-C	Consumption of oily fish improves triglycerides and HDL-C levels
**Abdelhamid et al., 2020** Cochrane Database Syst Rev	TC TG HDL-C LDL-C DBP SBP	SR and MA 30 RCTs 28 RCTs 30 RCTs 25 RCTs 15 RCTs 17 RCTs	Fish oil supplementation (mainly), dietary intake Comparing high versus low n-3 LC-PUFA intake	No effect on TC mean difference -0.01 (95% CI -0.05, 0.03) Decrease TG mean difference -0.24 (95% CI -0.31 to -0.16) Increase HDL-C, mean difference 0.03 (95% CI 0.0.1 to 0.05) No effect on LDL C mean difference 0.01 (95% CI -0.01 to 0.03)	Fish oil supplementation improves triglycerides and HDL-C levels
**Gao et al., 2020** Lipids in health and disease	Fasting glucose Fasting Insulin HbA1c HOMA-IR HDL-C TG TC LDL- C	SR and MA 12 RCTs among patients with T2DM (*n* = 820).	Fish oil supplementation Doses of EPA+DHA ranged from 0.3 g/day to 10.08 g/g	Fish oil decreased TG levels by -0.40 (95% CI -0.53 to -0.28) Fish oil increased HDL-C levels by 0.21 (95% CI 0.05 to 0.37) No effect on TC and LDL-C	Fish oil supplementation improves triglycerides and HDL-C levels

BP, blood pressure; SBP, systolic blood pressure; DBP, diastolic blood pressure; SR, systematic review; MA, meta-analysis; RCT, randomized controlled trial; TC, total cholesterol; TG, triglycerides.

**Table 5:** Characteristics of the studies evaluating fish intake and fish oil supplements and glucose, insulin, insulin sensitivity markers

Author	Outcomes	Type of study	Exposure	Results	Conclusion
**Alhassan et al., 2017** Atherosclerosis	Vascular risk factors. Primary outcomes included lipid biomarkers (TG, TC, HDL-C, LDL-C, VLDL-C, SBP, DBP, glucose, insulin, HOMA-IR, inflammation markers	SR and MA 14 clinical trials and RCTs *N* = 1,378 adults (>18 years) Mean follow up for 9 weeks (4–24 weeks)	Fish intake-the frequencies for consuming fish ranged from once a week to daily consumption, and portion size of oily fish (most commonly salmon), consumed on a given day ranged from 20 to 500 g.		No effect on insulin, glucose, or HOMA-IR
**Gao et al., 2017** Lipids in health and disease	Hyperinsulinemic-euglycemic clamp, Glucose clamp, HOMA-IR, Quantitative insulin sensitivity check index (QUICKI), Glucose toleranse	SR and MA 17 RCTs (*n* = 672)	Fish oil supplementation Doses of EPA+DHA 1 to 4 g/day	In pooled analysis, no effect on insulin sensitivity 0.17 (95% CI -0.15 to 0.48) Fish oil supplementation could benefit insulin sensitivity among people with at least one symptom of metabolic disorders 0.53 (95% CI 0.17 to 0.88) (no effect in T2D patients)	No overall effect of fish oil supplementation Subgroup analysis showed improved insulin sensitivity among people with metabolic disorders
**Gao et al.**, **2020** Lipids in health and disease	Fasting glucose Fasting Insulin (8 studies) HbA1c (10 studies) HOMA-IR (8 studies) HDL- C (15 studies) TG TC LDL-C	SR and MA 12 RCTs among patients with T2DM (*n* = 820).	Fish oil supplementation Doses of EPA+DHA ranged from 0.3 to 10.08 g/day	Fasting glucose SMD 0.13 (95% CI -0.03 to 0.28) fasting insulin SMD –0.00 (95% CI -0.22 to 0.21) HbA1c SMD 0.01 (95% CI -0.14 to 0.17) HOMA-IR SMD -0.02 (95% CI -0.19 to 0.14)	No effect on fasting glucose, fasting insulin, HbA1c and HOMA-IR

SBP, systolic blood pressure; DBP, diastolic blood pressure; SR, systematic review; MA, meta-analysis; RCT, randomized controlled trial; TC, total cholesterol; TG, triglycerides; SMD, standard deviation mean difference; HbA1c, glycosylated haemoglobin; HOMA IR, HOMA of insulin resistance.

**Table 6:** Characteristics of the studies evaluating fish intake, n-3 LC-PUFA intake and fish oil supplements and cancers

Author	Outcomes	Type of study	Exposure	Results	Conclusion
**Wu et al., 2012** Am J Med.	**Colorectal cancer**	MA 22 prospective cohort studies (*N* = 1,209,489 participants in total with 7,483 cases) and 19 case-control studies	Fish consumption, highest versus lowest	Fish consumption decreased the risk of colorectal cancer by 12% (summary OR, 0.88; 95% CI, 0.80–0.95). The pooled ORs of colorectal cancer for the highest versus lowest fish consumption in case-control studies and cohort studies were 0.83 (95% CI, 0.72–0.95) and 0.93 (95% CI, 0.86–1.01), respectively.	Findings suggest that fish consumption is inversely associated with colorectal cancer.
**Vieira et al., 2017** **Ann Oncol**	**Colorectal cancer**	MA & SR 11 studies included in the dose-response meta-analysis 10,356 cases	Fish consumption, highest versus lowest	An increase of 100 g/day of fish was associated with lower risk of colorectal cancer, RR 0.89(95% CI 0.80–0.99)	The inverse association for fish intake showed low credibility because the results were mainly driven by one study in the analysis (40% weight).
**Schwingshackl et al., 2018** Int. J. Cancer	**Colorectal cancer**	MA & SR 21 studies examined (cohort studies, case–cohort studies, follow-up of RCTs and nested case–control studies) 19,996 cases	Fish consumption, highest versus lowest	Overall risk estimates of 0.95 (0.87 to 1.03) per 100 grams fish per day There was no evidence of a nonlinear dose-response (15 studies included)	No statistically significant association was observed between fish intake and risk of colorectal cancer. However, an inverse association was observed for studies conducted in Europe, for long-term studies, and studies with 1,000 cases or more.
**Lovegrove et al., 2015** Int J Clin Pract	**Prostate cancer**	SR 37 studies in total (case-control and cohort) 31 on PCa risk 8 on aggressiveness 3 on mortality 495,321 participants in total	Fish intake Fish oil intake	Overall, 10 studies considering PC-risk found significant inverse trends with fish and fish-oil intake. Three studies investigating fish consumption and PC-mortality identified a significantly reduced risk. Multivariate-OR (95% CI) were 0.9 (0.6–1.7), 0.12 (0.05–0.32) and 0.52 (0.30–0.91) at highest fish intakes.	Total fish and fish oil intake was not associated with prostate cancer risk but may be implicated in lower mortality risk.
**Aucoin et al., 2017** Integrative Cancer Therapies	**Prostate cancer**	SR 11 cohort studies on primary prevention (17 publications) 13 publications on diet, one on supplement and 3 on both	Fish intake Fish oil	Of the analyses assessing prostate cancer incidence prospective, 12 did not show a statistically significant association. Five analyses showed a significant association between increased intake of fish-derived omega-3 fatty acids and decreased prostate cancer incidence.	Insufficient evidence to suggest a relationship between fish-derived omega-3 fatty acid and risk of prostate cancer. An association between higher omega-3 intake and decreased prostate cancer mortality may be present, but more research is needed
**Zheng et al., 2013** BMJ	**Breast cancer**	MA & SR 21 cohort studies 11 studies on fish intake 687,770 participants 13,323 cases 17 studies on marine n-3 PUFA 527,392 participants 16,178	Fish intake n-3 LC-PUFA from diet or as tissue biomarker	High fish intake yielded overall risk estimate of 1.03 (0.93–1.14) High intake of n-3 LC-PUFA from diet yielded overall risk estimate of 0.86 (0.78–0.94)	High intake of n-3 LC-PUFA is associated with 14% reduction in risk of breast cancer
**Wu et al., 2016** Nutrients	**Breast cancer**	MA 18 cohort studies 914,451 participants 19,400 cases	Fish intake (highest vs. Lowest and dose-response)	The summary RR for highest versus lowest was 1.04 (95% CI 0.97 to 1.12) Dose-response analysis showed that the summary RR per 120 g/day was 1.07 (95% CI 0.94 to 1.21)	A null association was observed for fish intake and risk of breast cancer.
**Kazemi et al., 2021** Adv Nutr	**Breast cancer**	SR and MA Included were cohort, case-cohort, nested casecontrol studies, and follow-up studies of randomized controlled trials 17 studies 28,818 cases	Fish intake (dose-response)	Dose-response analysis showed that the summary RR per 100 g/day increase was 1.0 (95% CI 0.93 to 1.08)	A null association was observed for fish intake and risk of breast cancer.

* Selection of topics for systematic reviews for the NNR2022 project

RR, Relative risk/Risk ratio; PUFA, polyunsaturated fatty acids; PCa, prostate cancer; SR, systematic review; MA, meta-analysis; RCT, randomized controlled trial; OR, Odds ratio; RR, Relative risk/Risk ratio.

**Table 7:** Characteristics of the studies evaluating fish intake, n-3 LC-PUFA intake and fish oil supplements and risk of metabolic syndrome

Author	Outcomes	Type of study	Exposure	Results	Conclusion
**Karimi et al., 2020** Nutrition, Metabolism, and cardiovascular diseases	Metabolic syndrome	SR and MA 10 cross-sectional studies 6 cohort studies	Fish intake, highest versus lowest	An inverse association between fish intake and risk of MetS OR 0.80 (95% CI 0.66 to 0.96) in cohort studies No association between fish intake an risk of MetS OR 0.85 (95% CI 0.70 to 1.02) in cross-sectional studies	
**Kim et al., 2021** Nutrients	Metabolic syndrome	7 cross-sectional studies 2 cohort studies	Fish intake or n-3 LC-PUFA, highest versus lowest intake	RR 0.71 (95% CI 0.58 to 0.87) highest versus lowest fish intake and RR 0.94 (95% CI 0.90 to 0.98) one serving/week increment. RR 0.58 (95% CI 0.48 to 0.70) highest versus lowest n-3 FA intake and RR 0.88 (95% CI 0.85 to 0.92) every 100 mg/day increment. OR 0.85 (95% CI 0.59 to 1.22) and n-3 LC-PUFA intake OR 0.94 (95% CI 0.79 to 1.12)	An inverse association between fish consumption and risk of metabolic syndrome in cohorts An inverse association between n-3 LC-PUFA intake and risk of metabolic syndrome n cohorts No association between fish intake and n-3 LC-PUFA intake and risk of metabolic syndrome in cross sectional studies

SBP, systolic blood pressure; DBP, diastolic blood pressure; SR, systematic review; MA, meta-analysis; RCT, randomized controlled trial; OR, Odds ratio; RR, Relative risk/Risk ratio.

**Table 8:** Characteristics of the studies evaluating fish intake and fish oil supplements and C-reactive protein

Author	Outcomes	Type of study	Exposure	Results	Conclusion
**Alhassan et al., 2017** Atherosclerosis	Vascular risk factors. Primary outcomes included lipid biomarkers (TG, TC, HDL-C, LDL-C, VLDL-C, SBP, DBP, glucose, insulin, HOMA-IR, inflammation markers	SR and MA 14 clinical trials and RCTs *N* = 1,378 adults (>18 years) Mean follow up for 9 weeks (4–24 weeks)	Fish intake-the frequencies for consuming fish ranged from once a week to daily consumption, and portion size of oily fish (most commonly salmon), consumed on a given day ranged from 20 to 500 g.		No effect on CRP
**Lin et al**., **2016** Lipids in Health and Disease	Inflammatory markers including IL-2, IL-6, TNFa, CRP	8 RCTs (*n* = 955), patients with T2DM adults (> 18 years) duration of RCT from 6 to 12 weeks,	Fish oil providing a mix of EPA+DHA or pure EPA or DHA. A minimum daily dose of EPA+DHA was 2 g/day and max dose was 6 g	CRP was significantly decreased in the n-3 LC-PUFA group compared to control group SMD 1.90, 95% CI 0.64 to 3.16, *P* = 0.003. Test for heterogeneity was significant (*I* ^2^ = 98%, *P* < 0.00001)	Fish oil reduces CRP, but large variation in responses

SBP, systolic blood pressure; DBP, diastolic blood pressure; SR, systematic review; MA, meta-analysis; RCT, randomized controlled trial; SMD, standard deviation mean difference; TC, total cholesterol; TG, triglycerides; CRP, C-reactive protein.

## Fish intake in Nordic and Baltic countries

Many food-based recommendations include fish as part of a healthy diet, and it is common to suggest 2–3 servings per week (22). The Nordic, and some of the Baltic, countries give similar recommendations regarding fish intake as a part of healthy dietary pattern (Table 9). The health effects from consuming fish are partly due to the n-3 LC-PUFA content as well as the substitutional effect when fish and seafood are replaced by other sources of animal protein (23). Among the Nordic and Baltic countries, the highest fish consumption on average is in Norway and Iceland (Table 10). The lowest fish intake is in Estonia and Lithuania, based on data collected in 2010 (Iceland, Norway, and Sweden), 2011 (Denmark), 2014 (Estonia), 2017 (Finland), 2019 (Lithuania), and 2020 (Latvia) (1).

**Table 9:** Recommendations on fish intake in the Nordic and Baltic countries

Country	Recommendation
Denmark	350 g/week (2 meals p/week and as topping on bread) -thereof 200 g fatty fish
Finland	2–3 fish meals p/week
Iceland	300 to 450 g/week -thereof 150 g fatty fish 2–3 meals p/week and as topping on bread
Norway	300 to 450 g/week -thereof at least 200 g fatty fish 2–3 meals p/week and as topping on bread
Sweden	2–3 fish meals p/week Important to vary the types of fish
Estonia	Eat less red meat, prefer fish and poultry
Latvia	Eat fish at least twice a week
Lithuania	-

Denmark: https://altomkost.dk/raad-og-anbefalinger/de-officielle-kostraad-godt-for-sundhed-og-klima/spis-mindre-koed-vaelg-baelgfrugter-og-fisk/

Finland: https://www.ruokavirasto.fi/globalassets/teemat/terveytta-edistava-ruokavalio/ravitsemus--ja-ruokasuositukset/sv/naringsrekommendationer_2014_web.pdf

Iceland: https://island.is/en/nutrition-recommendations

Norway: Kostrådene – Helsedirektoratet

Sweden: Livsmedelsverkets rapportserie

Estonia: Reccomendations avilable on FAO webpage – Food-based dietary guidlines

Latvia: Reccomendations avilable on FAO webpage – Food-based dietary guidlines

Lithuania: Not available on the FAO web page

**Table 10:** Average fish consumption in the Nordic and Baltic countries^a^

Country	Participants	Fish, g/week
Denmark 2011 (18–75 years)	Men (*n* = 1,464)	280
	Women (*n* = 1,552)	238
Finland 2017 (18–74 years)	Men (*n* = 780)	252
	Women (*n* = 875)	189
Iceland 2010 (18–80 years)	Men (*n* = 632)	385
	Women (*n* = 680)	266
Norway 2010 (18–70 years)	Men (*n* = 862)	553
	Women (*n* = 925)	392
Sweden 2010 (18–80 years)	Men (*n* = 792)	301
	Women (*n* = 1,005)	259
Estonia 2014 (18–74 years)	Men (*n* = 907)	203
	Women (*n* = 1,806)	161
Latvia 2020 (19–64 years)	Men (*n* = 470)	252
	Women (*n* = 541)	182
Lithuania 2019 (19–75 years)	Men (*n* = 1,348)	203
	Women (*n* = 1,562)	198

a Lemming and Pitsi (1).

In Denmark, Finland and Norway intake of fish is reported partly as raw weight and not as consumed as in the other countries.

## Health outcomes relevant for Nordic and Baltic countries

### Obesity and body weight

There is weak evidence for an association between fish intake on weight gain. The risk-benefit assessment by VKM, based on prospective cohort studies, found that the evidence of total fish intake on weight gain and risk of overweight in adults and in children is limited, with no conclusion (4) (Table 1). Concerning fish oil supplementation, a meta-analysis of six randomized controlled trials (RCTs) showed no difference in body weight reduction among children and adolescents (24) (Table 2). A meta-analysis of 21 RCTs showed no difference in body weight reduction among overweight and obese adults (25) (Table 2).

### Cardiovascular outcomes

There is strong evidence for a protective association between fish intake and CVDs. The VKM assessment (4) stated that total fish intake probably reduces the risk of coronary heart disease (CHD) and stroke incidence, and CVD, CHD, myocardial infarction (MI), and stroke mortality (Table 1). The evidence for any protective effect on incidence of CVD, MI, and heart failure was limited and suggestive. These findings are in line with the findings in a qSR by Rimm et al. (7) that concluded that fish consumption reduces the risk of cardiac death, CHD, and stroke (Table 1). One observed concern regarding fish consumption is the risk of adverse effect on atrial fibrillation, but the evidence is limited and suggestive (Table 1). Evidence is more limited on lean and fatty fish intake, most probably because of limited data (4). However, intake of n-3 LC-PUFA acids as supplements also shows protective effects on several CVD outcomes (4, 7, 14), and therefore species higher in these fatty acids may have a more protective effect. No effect of fish consumption on high blood pressure incidence was observed in three systematic reviews including cohort studies, case-cohort, and nested case-control studies (26), clinical trials and RCTs only (27) and cohort studies only (28) (Table 3). Fish oil supplementation showed a small reduction in blood pressure among hypertensive participants in two systematic reviews (29, 30), but no effect was observed in normotensive participants (29) (Table 3).

A meta-analysis of 14 clinical trials and RCTs found a reduction in triglycerides by 0.11 (95% confidence interval -0.18 to -0.04) mmol/L and an increase in HDL cholesterol (HDL-C) by 0.06 (95% CI 0.02 to 0.11) mmol/L after consumption of fatty fish (27) (Table 4). These data were also supported by two meta-analyses of 28 and 30 RCTs who found a reduction in triglycerides by 0.24 (-0.31 to -0.16) mmol/L and an increase in HDL-C by 0.03 (0.01 to 0.05) mmol/L after fish oil supplementation, respectively (31). A meta-analysis of 12 RCTs among T2D patients also found a reduction in triglycerides by 0.40 (-0.53 to -0.28) mmol/L and an increase in HDL-C by 0.21 (0.05 to 0.37) mmol/L (32) (Table 4). No effects on total cholesterol or LDL-C were observed in any of the meta-analyses.

### Type 2 diabetes

There is weak evidence for an association between fish intake and T2D. The VKM assessment concluded that the evidence for any effect of fish consumption on T2D risk and T2D mortality was limited and suggestive (4) (Table 1). A meta-analysis of 14 RCTs also showed no effects of fish intake on fasting insulin, glucose, or homeostatic model assessment for insulin resistance (HOMA-IR) (27) (Table 5). A meta-analysis of 17 RCTs showed no effect on insulin sensitivity by fish oil supplementation (33) (Table 5). In another meta-analysis of 12 RCTs, no effect of fish oil supplementation was observed on fasting insulin, hemoglobin A_1C_ (HbA1c), and HOMA-IR among T2DM patients (33) (Table 5).

### Cancers

There is weak evidence for an association between fish intake and cancers. The continuous update project (CUP) of the World Cancer Research Fund (WCRF) summarized the evidence between fish intake and risk of the above-mentioned cancer types in 2018 (18). The CUP project concluded that the evidence between high fish intake and lower risk of colorectal cancer (CRC) to be limited and suggestive (18, 34). This conclusion is in line with the risk-benefit analyses of fish consumption by VKM, which stated that the evidence between intake of fish and CRC was limited and suggestive (Table 1) (4). Three meta-analyses were identified through the literature search for CRC. Two suggested an inverse association between fish consumption and CRC. The third one, a meta-analysis published in 2018 by Schwingshackl et al. (35) found no association between fish intake and CRC. However, an inverse association was observed only for studies conducted in Europe, long-term studies, and studies with 1,000 cases or more (Table 6). In the study from 2012, a 12% risk reduction was observed for high fish intake and CRC. One of the search terms the author used was ‘fresh fish’, to avoid, for example, salted fish as an exposure (36). In the two meta-analyses for prostate cancer (37, 38) and the three meta-analyses for breast cancer (39–41), the results were insufficient for any conclusion to be made, although some of the meta-analyses focused only on fish intake and others also on n-3 LC-PUFA intake (Table 6).

### Neurodevelopment in children

There is weak evidence for an association between both maternal exposure to fish consumption during pregnancy and exposure to fish in childhood and neurodevelopment in children. VKM stated that the evidence for a beneficial effect on neurodevelopment of children, both maternal exposure to fish consumption during pregnancy and children’s exposure to fish in childhood, was limited and suggestive (4) (Table 1). The US Dietary Guidelines Advisory Committee (DGAC) in the US concluded that there was moderate evidence for a favorable effect of seafood consumption during pregnancy on cognitive development in young children (12) (Table 1). The DGAC also concluded that there was limited evidence that seafood intake during pregnancy is associated favorably with measures of language and communication development, movement, physical development, and social-emotional and behavioral development in the child (12). Furthermore, no evidence was available to determine the relationship between maternal seafood intake during lactation and neurodevelopment in the child (12, 15) (Table 1). The DGAC concluded that there was insufficient evidence to determine a favorable relationship between seafood intake during childhood and adolescence and cognitive development in children and adolescents (15) (Table 1). Furthermore, they concluded that there was limited evidence for any beneficial effects of n-3 LC-PUFA supplementation during pregnancy and in children on cognitive development (16). This conclusion is also in line with the VKM report (4).

### Cognitive and mental health in adults

There is strong evidence for a protective association between fish intake and cognitive decline, but no strong evidence for mental health. Among adults, the risk-benefit analysis stated that the evidence for fish intake and cognitive decline in adults (e.g. dementia and Alzheimer’s) was probably protective (4) (Table 1). The evidence for an effect of fish intake on depression in adults and postpartum depression was limited and suggestive (4) (Table 1). The evidence for intake of n-3 LC-PUFA on cognitive function in adults was limited, there is no conclusion (4). This is in line with the qSR by Brainard et al., who stated that n-3 LC-PUFA supplementations do not protect older adults against cognitive decline (11).

### Asthma and allergy

There is weak evidence for an association between both maternal exposure to fish consumption during pregnancy and children’s exposure to fish in childhood and asthma and allergy in children. VKM (4) stated that the evidence for an effect of maternal intake of fish during pregnancy on asthma and allergy in the offspring was limited (Table 1). According to one systematic review and meta-analysis, fish oil supplementation during pregnancy and lactation may reduce the risk of allergic sensitization to eggs (8).

A *de novo* SR performed for the NNR2023 project concluded that intake of n-3 LC-PUFA supplements during pregnancy may reduce the risk of asthma and/or wheeze in the offspring, but the strength of evidence was low (9). There was inconclusive evidence for the effects of n-3 LC-PUFA supplementation during pregnancy for other asthma and allergy outcomes, as well as for supplementation during lactation or infancy (9).

### Metabolic syndrome

There is evidence for an association between fish intake and metabolic syndrome. In a meta-analysis of six cohort studies, an inverse association between fish intake and risk of metabolic syndrome was seen (OR 0.80; 95% CI 0.66 to 0.96) (42) (Table 7). In another meta-analysis of two cohort studies, an inverse association between fish intake and risk of metabolic syndrome was also seen (RR 0.71; 0.58 to 0.87). This was also observed for n-3 LC-PUFA intake (RR 0.58; 0.48 to 0.70) (43) (Table 7). In a meta-analysis of 10 cross-sectional studies, no association between fish intake and risk of metabolic syndrome was observed (42) (Table 7). An association between fish intake or n-3 LC-PUFA intake and risk of metabolic syndrome was also not observed in a meta-analysis of seven cross-sectional studies (43) (Table 7).

### Birth outcomes

There is strong evidence for a protective association between maternal fish consumption during pregnancy and birth outcomes. VKM stated that the evidence for reducing the risk for preterm birth and low birth weight was probable (Table 1) (4). n-3 LC-PUFA supplementation studies do not show strong evidence for a protective association between maternal exposure to supplementation and reduced risk for preterm birth, but support the conclusion for low birth weight (17).

### Total mortality

There is strong evidence for a protective association between fish intake and total mortality. VKM stated that the evidence for all-cause mortality and fish intake was probably protective (Table 1) (4).

## Mechanisms

Among the important nutrients in fish, the mechanisms of the n-3 LC-PUFA EPA and DHA, are well described. These fatty acids are incorporated into the phospholipid bilayer of cell membranes in the body, and can affect the membrane fluidity and the function of cell-signaling receptors in the membrane of for example, cardiac, immune, and brain cells (7). DHA is an essential component of the phospholipid bilayer of brain cells, and via this mechanism, DHA may affect different neurotransmitter systems important for normal brain function and development (44–46).The n-3 LC-PUFA can also act as ligands for transcription factors and regulate genes important in lipid metabolism and inflammation (47). Moreover, n-3 LC-PUFA may have anti-inflammatory function by being converted to eicosanoids, which are signaling molecules affecting several cells involved in a wide range of processes including immune function (48). The n-3 LC-PUFA can prevent the conversion of arachidonic acid (an n-6 LC-PUFA) into pro-inflammatory eicosanoids by serving as an alternative substrate for cyclooxygenases or lipoxygenases. In addition, inflammation-resolving mediators are derived from n-3 LC-PUFA. Although experimental *in vitro* and animal studies have shown anti-inflammatory effects of n-3 LC-PUFA, limited evidence in humans exist. In the meta-analysis by Alhassan etal. no effect of fish intake and fish oil supplementation was found on the levels of C-reactive protein in 14 clinical trials and RCTs in adults (27) (Table 8). In a meta-analysis by Lin et al., which included eight RCTs with patients with T2D, a significant decrease in C-reactive protein was observed, but there were large variations in the effect after fish oil supplementation (49) (Table 8). In addition to n-3 LC-PUFA, proteins in fish may explain the health beneficial effects of fish consumption. Fish-derived peptides contain bioactive amino acids that may influence hypertension and the lipid profile (50).

### Fish in food based dietary guidelines

Fish plays a substantial part of the Nordic diet (1), and contains important nutrients for optimal function of the body. The Nordic, and some of the Baltic, countries give similar recommendations regarding fish intake as a part of healthy dietary pattern (Table 9). Based on current evidence, total fish consumption is beneficial for prevention of CHD, stroke, metabolic syndrome, and cognitive decline, of mortality from CVD, CHD, MI and stroke, and of total mortality. Many CVD outcomes and child neurodevelopment (both maternal exposure to fish consumption during pregnancy and children exposure to fish in childhood) also have limited, suggestive beneficial effects due to an inadequate number of studies, inconsistency in results, risk of bias in classification of exposures, and heterogeneity of outcome assessments. There is also insufficient evidence currently available to determine the relationship between seafood consumption during childhood and adolescence and risk of developing CVD (13). Since both CVD and cognitive decline take years to develop, and there are suggestive beneficial effects of fish intake during childhood, it is important to give advice about fish consumption throughout life course.

In future studies on fish intake and health outcomes covered in this scoping review, more focus needs to be on repeated fish exposure assessments throughout the life course, as well as better documentation of fish types (lean or fatty fish), possible pollution in different areas, preservation, and cooking methods.

### Advice for setting food-based dietary guidelines

Strong evidence supports the recommendation to consume fish. The evidence is strongest for total fish intake and health outcomes related to CVD and cognitive decline (Alzheimer’s and dementia), which are diseases affecting many people, with serious consequences for those who are affected and the society. Since the biological mechanisms for n-3 LC-PUFA support a protective effect, and intake of n-3 LC-PUFA supports some of the CVD outcomes, this implies that intake of fatty fish is of importance, and that we should continue to advice people to eat fatty fish as part of a healthy diet recommended in the FBDGs. Despite that fatty fish may contain contaminants, the benefit of eating fish outweighs the risks that contaminants may cause (4). The differences between the countries in fish intake may be related to accessibility, recommendations, and possible pollution in special areas. It is therefore advisable that each country continue to keep their FBDGs for fish intake, adjusting for variability of available fish and areas of contamination.
